# Supplementary material for: Three-dimensional holographic imaging of incoherent objects through scattering media
Source: Nat Commun. 2025 Nov 26;16:11653. doi: 10.1038/s41467-025-66626-7 (PMC12748562; doi:10.1038/s41467-025-66626-7)
Supplement: Supplementary file 1 — Supplementary Information [file 41467_2025_66626_MOESM1_ESM.pdf]

Contents

Supplementary sections:

1. Shared distortion in scattered fields
2. Impact of virtual medium expression on image formation
3. Image correction for 3D objects

Supplementary figures:

Figure S1. Positional accuracy in 3D reconstruction

Figure S2. Numerical simulation of imaging 3D incoherent objects

Figure S3. Field correlation before and after unitary transformation

Figure S4. Local field correlation

Figure S5. Change in field correlation with axial position

Figure S6. Experimental setup

1 Shared distortion in scattered fields

Equation (2) of the main text shows that scattered fields from two neighboring sources are maximally correlated at a specific plane after adjusting wavefront tilt. This allows one field to be expressed in terms of the other:

$$\mathcal{E}_n(\mathbf{r}) \propto \mathcal{E}_m(\mathbf{r})\, e^{-i\Delta\mathbf{k}_{opt}\cdot\mathbf{r}} + M_n, \tag{S1}$$

where M_n is a small term uncorrelated with $\mathcal{E}_m$. The scattered field can be written as the product of the field in the absence of scattering, $h_n(\mathbf{r})$, and a distortion, $S_n(\mathbf{r})$, such that $\mathcal{E}_n(\mathbf{r}) = h_n(\mathbf{r})\, S_n(\mathbf{r})$. Substituting into the relation above gives:

$$h_n(\mathbf{r})\, S_n(\mathbf{r}) \propto h_m(\mathbf{r})\, e^{-i\Delta k_{opt}\cdot r} S_m(r) + M_n. \tag{S2}$$

Using $h_n(\mathbf{r}) = h_m(\mathbf{r})\, e^{-i\Delta\mathbf{k}_{opt}\cdot\mathbf{r}}$ and dividing by h_n, we obtain

$$S_n(\mathbf{r}) \propto S_m(\mathbf{r}) + \frac{M_n}{h_n}. \tag{S3}$$

This shows the existence of a shared distortion in all wavefronts. We can express the average distortion across all sources as

$$S_o(\mathbf{r}) = \sum_n S_n(\mathbf{r}). \tag{S4}$$

When a scattered field is backpropagated through S_o, the scattering-free wavefront is recovered:

$$\mathcal{E}_n^*(\mathbf{r})\, S_o(\mathbf{r}) = |S_o(\mathbf{r})|^2 h_n^*(\mathbf{r}) + M', \tag{S5}$$

where M' is a noise term arising from the uncorrelated terms. Propagating this to the source plane produces a sharp focus at the source location with background noise. This result shows that the object image can be reconstructed by back-propagating all the wavefronts through a virtual medium that reflects the effect of S_o.

2 Impact of virtual medium expression on image formation

An ideal virtual scattering layer should cancel out the scattering effect in each fields, such that

$$\mathcal{E}_n^*(\mathbf{r})S(\mathbf{r}) \approx h_n^*(\mathbf{r}). \tag{S6}$$

Since the scattering effect is completely compensated, the fields propagating through the layer converges to the position of their corresponding sources. However, we can obtain similar results with the expression in Eq. (3), where the effect of the virtual layer is equivalent to:

$$\mathcal{E}_n^*(\mathbf{r})S(\mathbf{r}) \approx h_n^*(\mathbf{r})h_m(\mathbf{r}). \tag{S7}$$

Thus, the propagation in Eq. (4) introduces a shift in the image due to $h_m^*(\mathbf{r})$, compared to the ideal scattering layer. To be specific, the corrected field in Eq. (S2) can be rewritten using Fresnel diffraction theory as

$$\mathcal{E}_n^*(\mathbf{r})S(\mathbf{r}) \propto \exp\left[\frac{\pi i}{\lambda}\left\{\left(\frac{1}{z_m} - \frac{1}{z_n}\right)|\mathbf{r}|^2 + 2\left(\frac{\mathbf{r}_n}{z_n} - \frac{\mathbf{r}_m}{z_m}\right)\cdot\mathbf{r} + C_n\right\}\right], \tag{S8}$$

where $\mathbf{r}_n = (x_n, y_n)$ represents the transverse coordinates, z_n is the axial coordinate of the n-th source, and C_n is a constant phase. If the sources are placed at the same depth, $z_n = z_m$, the expression simplifies to

$$\mathcal{E}_n^*(\mathbf{r})S(\mathbf{r}) \propto \exp\left[\frac{2\pi i}{\lambda z_m}(\mathbf{r}_n - \mathbf{r}_m)\cdot\mathbf{r} + C_n\right], \tag{S9}$$

whose propagation according to Eq. (4) forms a focus at $\mathbf{r} = (\mathbf{r}_n - \mathbf{r}_m)/\lambda z_m$ and $z = 0$. This result shows that the expression in Eq. (3) shifts the image, placing m-th source always at the origin. For general 3D objects, the image of n-th source appears at $\mathbf{r} = \mathbf{r}_n/\lambda z_n - \mathbf{r}_m/\lambda z_m$ and at $z = 4\pi^2/\lambda^2(z_n^{-1} - z_m^{-1})$. This corresponds to a scaling and translation of images at varying depths, similar to single-lens imaging.

3 Image correction for 3D objects

The image formation based on Eq. (S8) and Eq. (4) positions m-th source on the optic axis at an infinite distance from the virtual layer. This is analogous to single-lens imaging formation, where the magnification and position of an image varies depending on the position of an object. To comensate these effects, we need to know the position of the m-th source, specifically $\mathbf{r}_m$ and z_m, as shown in Eq. (S3). We can estimate these values by analyzing the m-th scattered field $E_m(\mathbf{r})$. The axial position of m-th source is determined by numerically propagating $E_m(\mathbf{r})$ and identifying the depth z at which the envelope of the speckle pattern is narrowest. The transverse position $\mathbf{r}_m$ is estimated as the center of this envelope. With the estimated $\mathbf{r}_m$ and z_m, the image $I(\mathbf{r}, z)$ in Eq. (4) can be adjusted to $I(\mathbf{r}_{adj}, z_{adj})$, where

$$z_{adj} = \left(\frac{1}{z_m} + \frac{\lambda^2 z}{4\pi^2} \right)^{-1}, \tag{S10}$$

and

$$\mathbf{r}_{adj} = z_{adj} \left(\lambda \mathbf{r} + \frac{\mathbf{r}_m}{z_m} \right). \tag{S11}$$

We note that while estimating the source position based solely on scattered fields may not be perfect, it is sufficient for the image correction. This is because the error in depth estimation, δz_m, has a negligible effect on the final image when $\delta z_m \ll z_m^2$. Similarly, the error in transverse position estimation, $\delta \mathbf{r}_m$, remains insignificant if $|\delta \mathbf{r}_m| \ll z_m$.

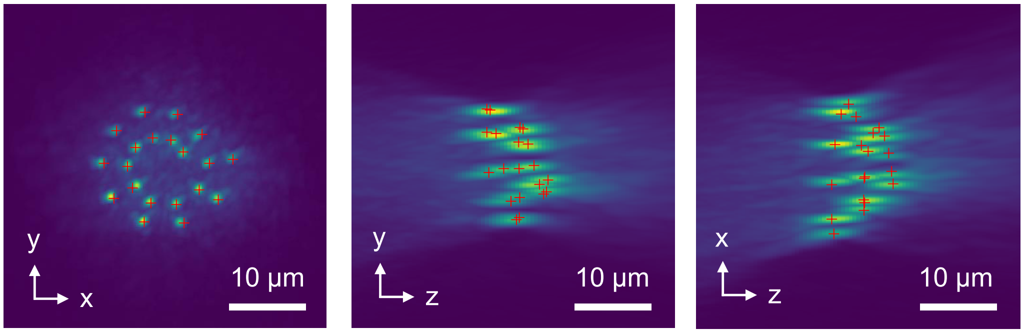

Figure S1. **Positional accuracy in 3D reconstruction**: The expected source positions are marked with red plus signs overlaid on the MIP images in Fig. 4d. The source positions are rotated by 7 degrees relative to the x-axis to account for the viewing angle mismatch between the illumination and detection setups.

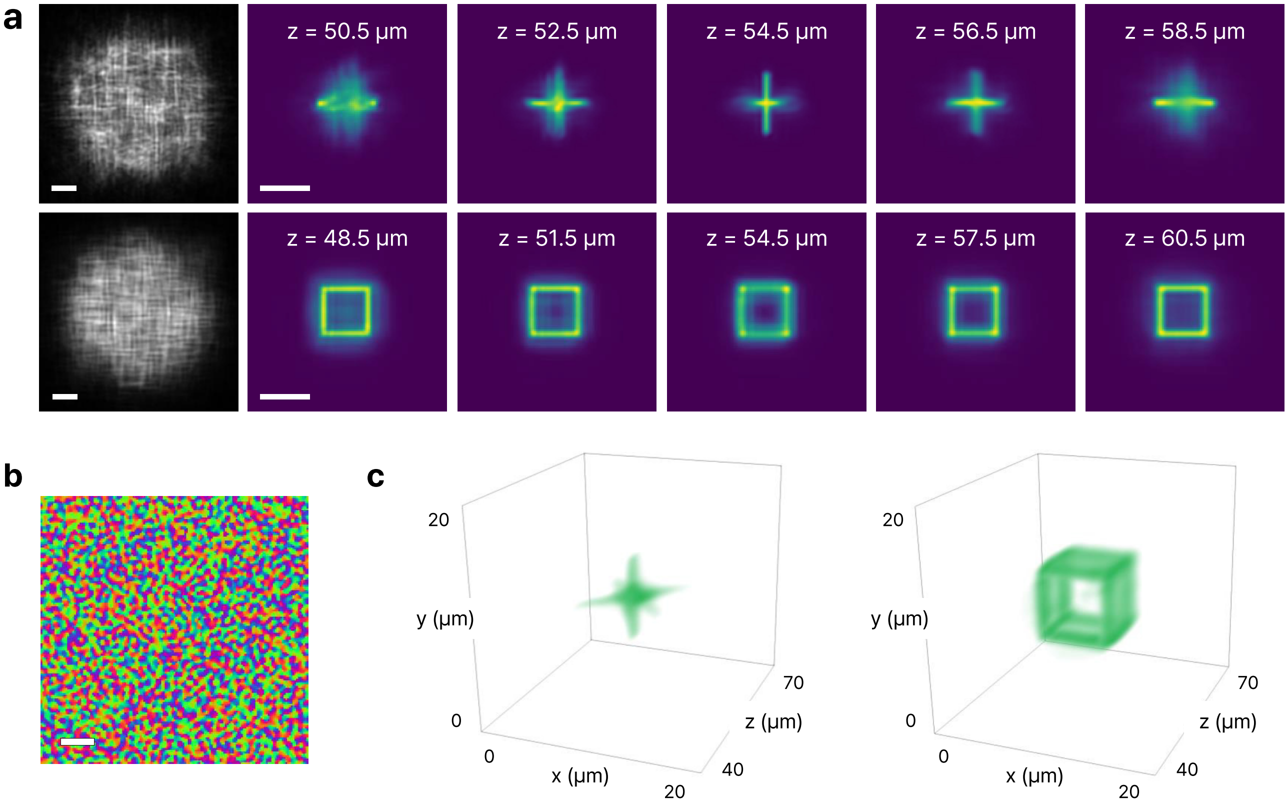

Figure S2. **Numerical simulation of imaging 3D incoherent objects**: **a**, Speckle images measured at the camera (leftmost column) and the reconstructed images at various depths. The simulated incoherent sources were arranged in the shape of three orthogonal lines and the outline of a cube. Scale bars represent 5 μm. **b**, Phase profile of the simulated scattering layer. **c**, 3D images (rendered) obtained using the proposed method.

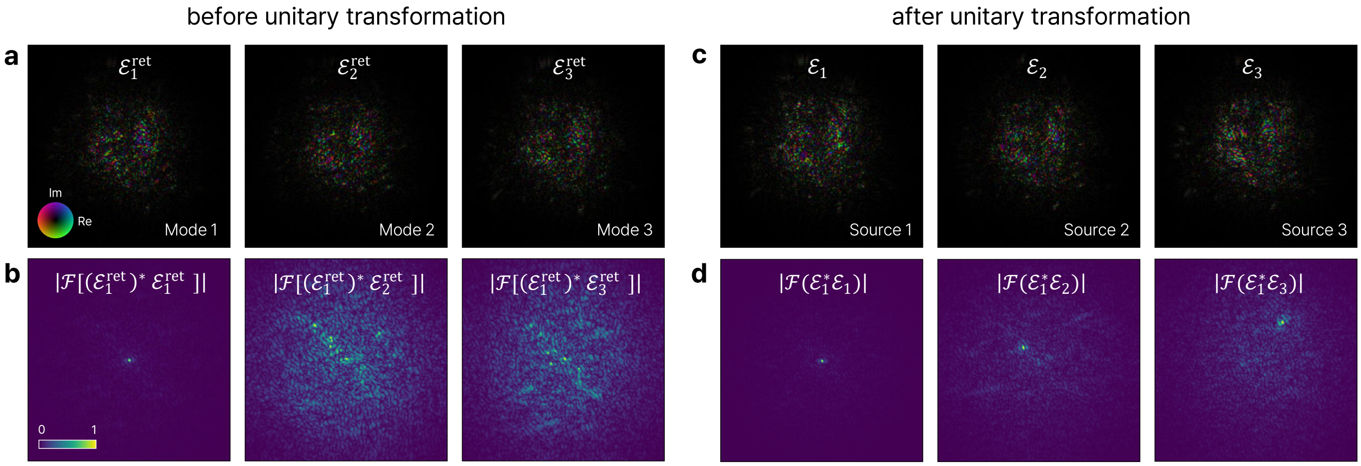

Figure S3. **Field correlation before and after unitary transformation**: **a**, Retrieved scattered fields (first three shown), representing mutually incoherent modes of fluorescence. **b**, Pairwise comparison of wavefronts of (a) at to correlation plane, using $\mathcal{E}_1^{\mathrm{ret}}$ as a reference. No evident correlation is observed between different fields. **c**, Scattered fields following the unitary transformation (first three shown). **d**, Pairwise comparison of (c) using $\mathcal{E}_1$ as a reference. Here, relative wavefronts exhibit clear correlation, characterized by linear phase ramps.

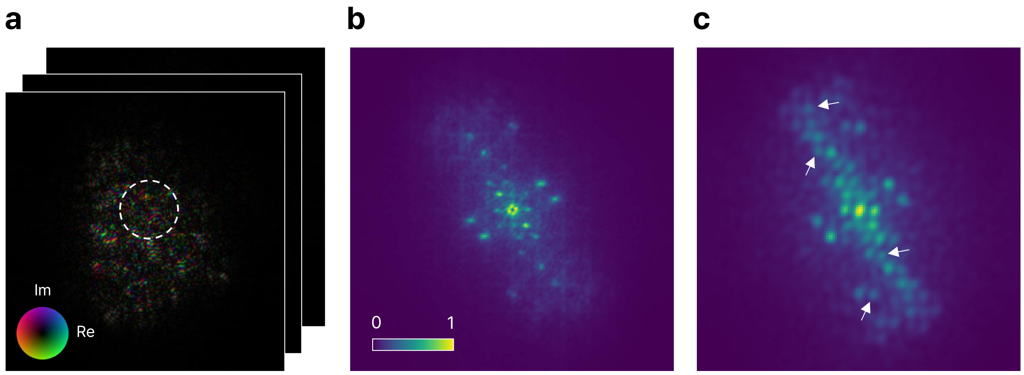

Figure S4. **Local field correlation**: **a**, Scattered fields from a 3D fluorescent object (shown in Fig. 3) at the correlation plane. **b-c**, The wavefront correlation $\Gamma(\mathbf{r}, z)$ of (a) using the entire field of view (b) and using a smaller region of interest (c), indicated by the dashed circle in (a). As the local relative wavefronts are approximated as linear ramps, the 2D wavefront correlation reveals additional peaks corresponding to sources at different depths, highlighted by white arrows).

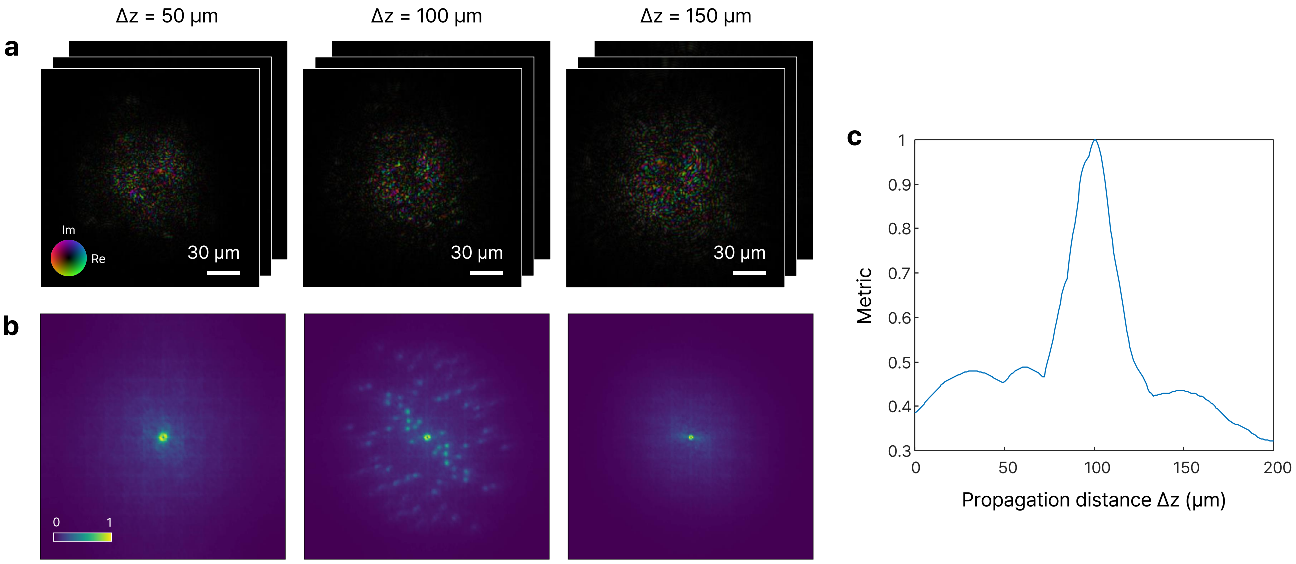

Figure S5. **Change in field-correlation with axial position**: **a**, Representative numerically propagated retrieved fluorescence fields. **b**, The wavefront correlation $\Gamma(\mathbf{r}, z)$ (Eq. 6) at different depths. **c**, Maximum values of $\Gamma(\mathbf{r}, z)$, used to determine the position of the correlation plane; $\hat{z} = 100\mu m$.

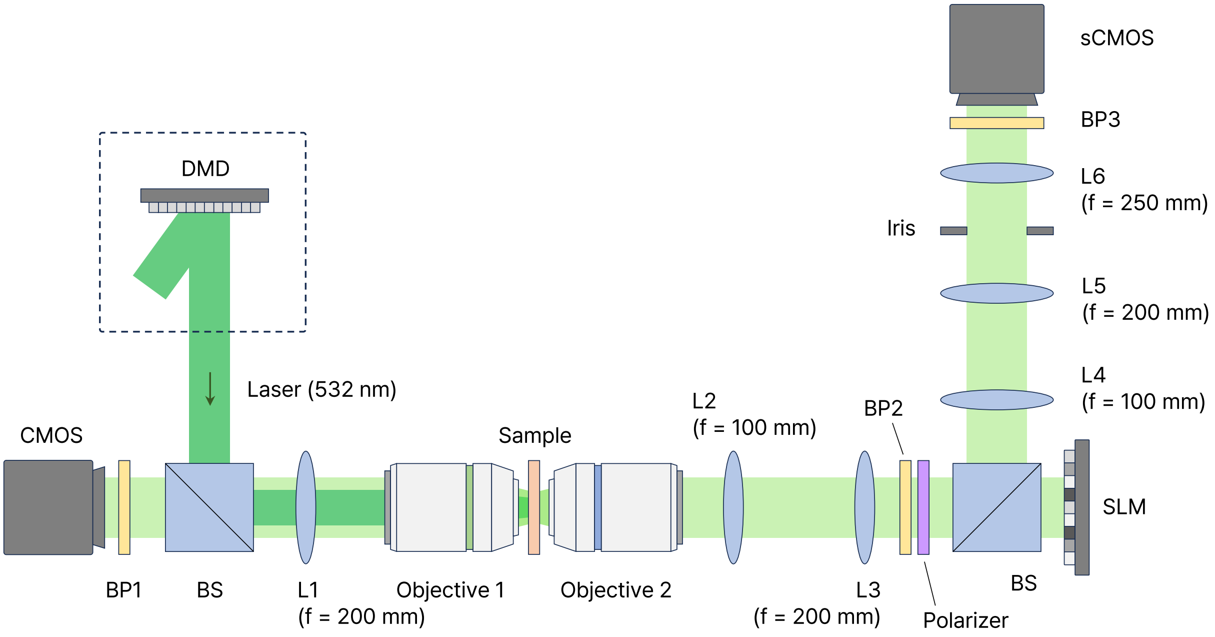

Figure S6. **Experimental setup**: L, lens; BS, beam splitter; BP, bandpass filter.
